# Surface Treatment and Bioinspired Coating for 3D-Printed Implants

**DOI:** 10.3389/fchem.2021.768007

**Published:** 2021-11-18

**Authors:** Junyi Liu, Nafisah Bte Mohd Rafiq, Lai Mun Wong, Shijie Wang

**Affiliations:** Institute of Materials Research and Engineering (IMRE), A*STAR (Agency for Science, Technology and Research), Singapore, Singapore

**Keywords:** surface treatment, bioactive coating, hydroxyapatite, bioglass, micro-arc oxidation, 3D printing, osseointegration, metallic implant

## Abstract

Three-dimensional (3D) printing technology has developed rapidly and demonstrates great potential in biomedical applications. Although 3D printing techniques have good control over the macrostructure of metallic implants, the surface properties have superior control over the tissue response. By focusing on the types of surface treatments, the osseointegration activity of the bone–implant interface is enhanced. Therefore, this review paper aims to discuss the surface functionalities of metallic implants regarding their physical structure, chemical composition, and biological reaction through surface treatment and bioactive coating. The perspective on the current challenges and future directions for development of surface treatment on 3D-printed implants is also presented.

## Introduction

In recent years, three-dimensional (3D) printing, also known as additive manufacturing (AM), has become an apparent choice for manufacturing technology. The manufacturing process is a bottom–up approach where raw materials are deposited layer-by-layer into a 3D object (Rayna and Striukova, 2016). The concept of 3D printing was first originated as rapid prototyping around the mid-1980s by Charles Hull (Bagaria et al., 2018). The first commercial 3D printing started with plastic, and by the mid-1990s metals had gained similar commercialization use (Duda and Raghavan, 2016). The ability to save cost, design complex shapes, and reduce waste are encouraging engineers and designers to tap into this technological capability (Vaz and Kumar, 2021). In addition, the new focus is currently channeling production of a new product design rather than choosing between AM and traditional manufacturing. 3D printing enables an effective buy-to-fly ratio reaching to equivalence, whereas conventional methods observed a 20-fold increase. A significant variation is observed due to raw materials cost, manufacturing processes, and other manufacturing-related logistics (Duda and Raghavan, 2016). This promising realization of 3D printing has since been implemented in production lines, particularly in automotive, aerospace, and medical industries, as recorded in many publications (Alison et al., 2019; Ni et al., 2019; Capasso et al., 2020).

Besides the promising attributes of 3D printing, a drawback is also commonly identified and associated with its undesirable esthetic build. Consequently, suffering from mechanical malfunction and lack of stability leads to poor performance across wide medical applications. Henceforth, this review serves to understand the various treatment methods from a biomedical point of view, mainly through understanding: 1) implant substrate parametrics such as surface uniformity, topography, wettability, and porosity, and 2) the integration of bioactive molecules and growth factor receptors to encourage key cellular activities.

Surface treatment is commonly coupled together with bioactive coating to achieve long-term implant stability, biocompatibility, and antibacterial surface protection. Despite many surface modification methods being discovered over the last decade, with many reviews translating experimental results into a limited set of stand-alone methods, there is no concise observed relationship between the range of biomedical metallic materials and surface modification methods. This, therefore, questions the applicability of metallic materials in performing and achieving similar outcomes across various surface methods, and likewise in the reverse. This paper also aims to widen the opportunity for cross-functional coating in maximizing the likelihood of implant survival.

### 3D Printing Usage in Medical Application

**FIGURE 1:**
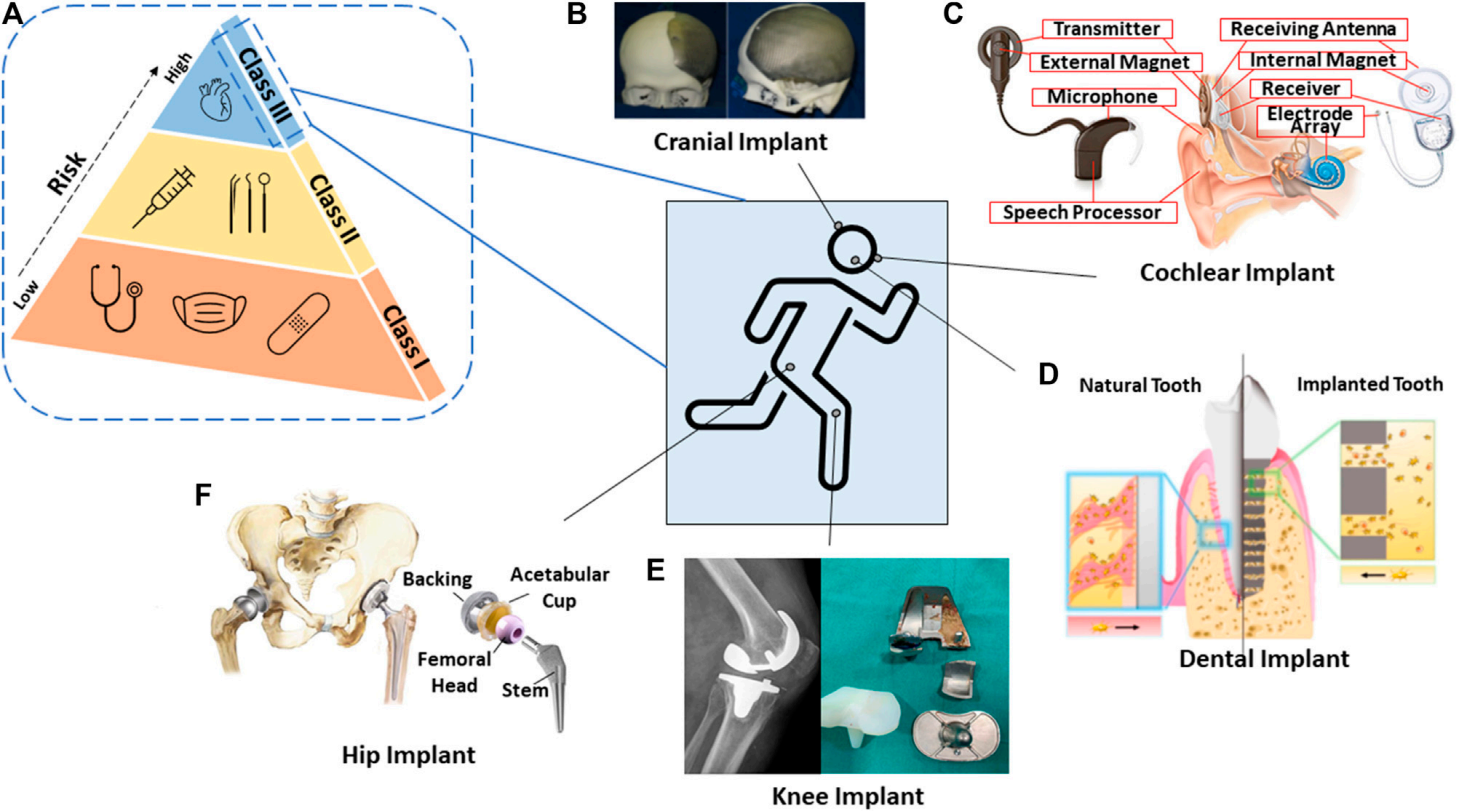
Schematic diagram of the **(A)** risk associated within FDA-classified medical devices; and some graphics of 3D-printed biomedical applications in the human body system. **(B)** Cranial implant (Jardini et al., 2016). **(C)** Cochlear implant (Brand et al., 2014). **(D)** Dental implant (Yin et al., 2021). **(E)** Knee implant (Chithartha et al., 2020). **(F)** Hip implant (Mattei et al., 2011).

Medical material development has been prominent over the past three decades. Throughout this time, the most notable materials used were ceramics due to their similar constituents to the human body, for example, the bone-regeneration constituent containing calcium phosphate that can be easily reproduced (Best et al., 2008). Ceramic material is categorized as bioinert or bioactive and shows strong interfacial bonding to the host tissue and osseointegration capability compared to other material classification. When it comes to medical device classification, it is subdivided into three categories implemented by the Food and Drug Administration (FDA). These are Class I, II, and III, with the latter being described as high risk to the human body, as illustrated in Figure 1; therefore, safety measures are adopted and required to be approved prior to mass release to the public (Johnson, 2016).

In the past, there were no systematic guidelines being introduced for materials, such as metallic, ceramic, and polymer, that could be considered for commercial biomedical devices. This resulted in more than 700 deaths and 10,000 patient injuries (Dhruva and Redberg, 2013). As the framework became gradually implemented, likewise for 3D-printed devices, similar validation and specific requirements such as design and quality control strategies came to be utilized, resulting in controlled output and consistent production of the devices (Morrison et al., 2015). Within the European Union (EU), custom-made implants, which are intended to penetrate beneath the epithelial layer such as contact lenses or devices embedded inside the body, require CE marking and have to abide by regulation (Aimar et al., 2019).

To fabricate parts of complex and intricate structures to fit nicely on the patient, for example, skull reconstruction, traditional ways involving mesh implant insertion limit dimensional precision and structural integrity. Therefore, 3D printing has an upper hand when it comes to designing complex structures which allow successful integration to the host tissue. Particularly, in 3D-printed dental implants, the flexibility of tuning micropore channel architecture consequently facilitates signaling molecules to be activated at specific sites. This is useful in recruiting cells and maintaining alveolar height in a dental implant for better healing ability (Yin et al., 2021). The absence of surface treatment in the earlier period of pacemaker implant invention that revolves around metallic material, such as stainless steel, caused challenging mechanical fracture to its “pacemaker lead,” eventually causing declining electrical stimulus. Many attempts have been made to replace the metallic alloy component, but no sign of improvement has been observed aside from more issues, including those related to corrosion (Joung, 2013). There are many significant benefits surrounding 3D printing as mentioned in many articles from its customization (Attarilar et al., 2020) to being cost effective (Schubert et al., 2014), and being ready-built in several hours (Mertz, 2013). Specifically, healthcare professionals use 3D printing to engage with patients via software, such as MIMICS or MeDraw, without requiring engineering expertise to develop and analyze models (Dai and Xu, 2021). Through 3D printing, anatomical models serve to minimize design error on patients and provide extensive practice to physicians prior to surgical procedures. As a result, this provides direct communication between physicians and patients to translate CT/MRI scans beyond a two-dimensional layout when treating life-threatening disease. This multidimensional function of 3D printing thus tackles medical issues effectively and efficiently while reducing the long waiting lists for treatment (Aimar et al., 2019). Another point that is not often emphasized or is overlooked is the open-source nature of the 3D printing files which allows vast collaboration among researchers and physicians more than just restrictive parameters published in scientific journals. The open-source database allows professionals to selectively tailor design dimensions according to the anatomical model of patients (Gross et al., 2014; Ventola, 2014). To a greater degree, open-source sharing of 3D models in the STL (Standard Tessellation Language or STereoLithography) file format has been proactively used in the immunology field, such as in the tiny replicas of microorganisms to visualize proteins and viruses (Coakley et al., 2014). In short, the importance of 3D printing has extended across vast medical applications from operation planning, as instrument guides to implant devices, and even in the field of microorganisms (Dai and Xu, 2021).

**FIGURE 2:**
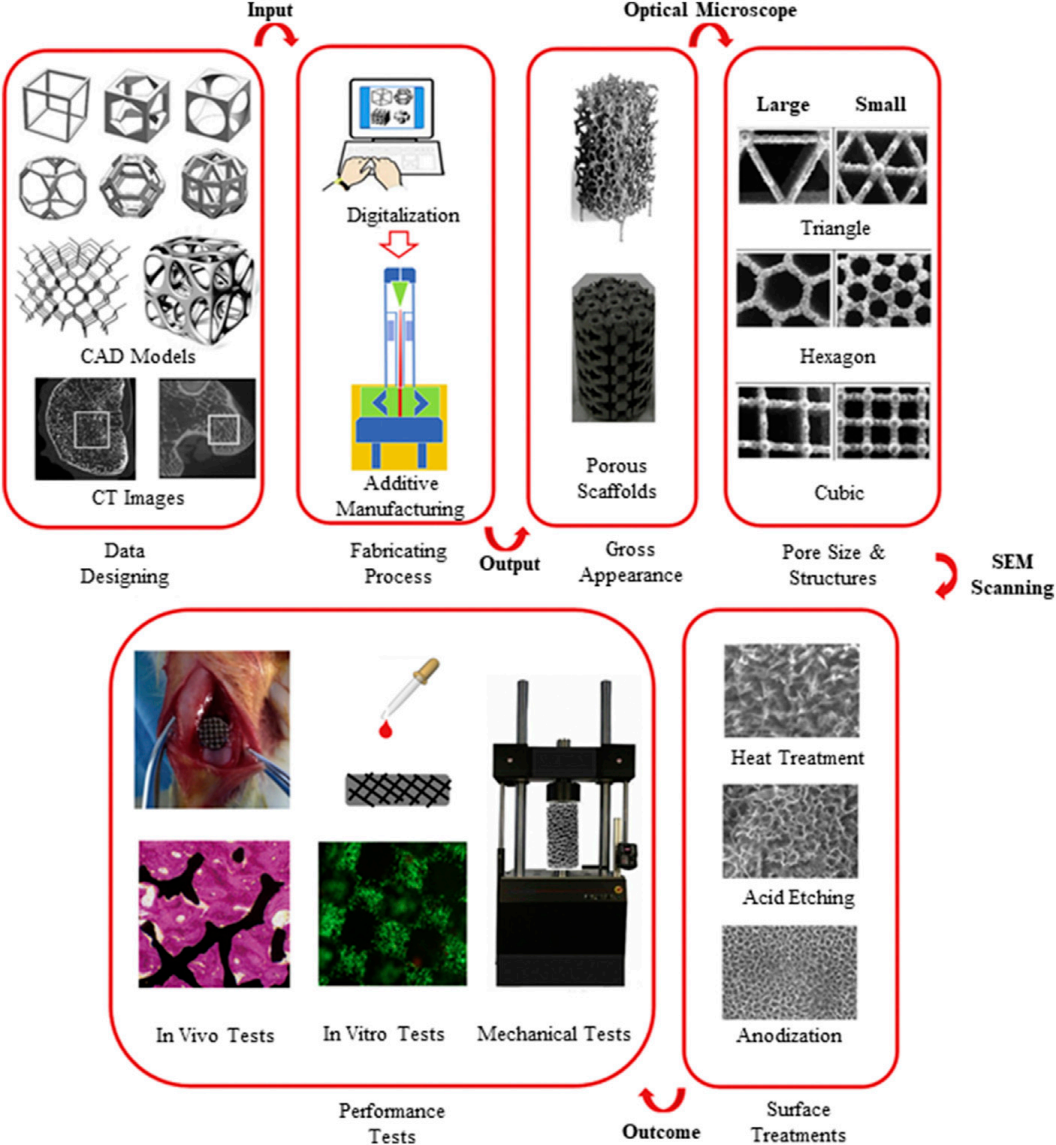
A step-by-step approach of design control and performance evaluation to achieve successful fabrication of a 3D-printed biomedical implant (Wang et al., 2017).

A typical procedure of a bottom–up 3D printing approach has been illustrated by Wang et al. (2017) (Figure 2). Particularly in orthopedic application, there are drawbacks in manufacturing porous implants using conventional methods which subsequently lead to implant failure due to lack of bone–implant integration. Depending on the final medical device customization and mechanical profile desired, a variety of surface modification techniques can be adopted, which are explored more in the Surface Modification Techniques section.

### Engineering Constraints within Metallic Biomaterials

Metallic bio-devices can be divided into degradable and nondegradable types. A degradable metallic material, such as magnesium alloy, has bioactive capability and interfacial interaction between implant and tissue. The ability to degrade is preferred for temporary implant support such as plates and screws in bone healing, thereupon eliminating the unwanted surgical risk and excessive cost of a second surgery (Gu et al., 2014). By contrast, a nondegradable metallic material is classified as bioinert through the formation of a tissue fibrous capsule which isolates the implant from the surrounding tissues; however, the process does not trigger adverse interference to the biological system to a certain extent (Daghighi et al., 2013). A suitable implant can be either bioinert or bioactive as long as it does not induce toxicity. In principle, a material serving as an implant in the human body must ensure biocompatibility to the host tissue. In the medical field, the commonly used materials for orthopedic application are comprised of titanium alloy (Xiu et al., 2016), cobalt chromium alloy, and stainless steel (Balazic and Kopac, 2007), which are also categorized as nondegradable materials.

The strength-to-weight ratio of titanium alloy and magnesium alloy demonstrates similar mechanical capability to the bone, consequently exhibiting good biological integration. However, when it comes to clinical capability, these alloys tend to suffer from accelerated corrosion rate and poor cell viability when in contact with human body fluids (Kazantseva, 2018; Zhang et al., 2019; Atrens et al., 2020).

**FIGURE 3:**
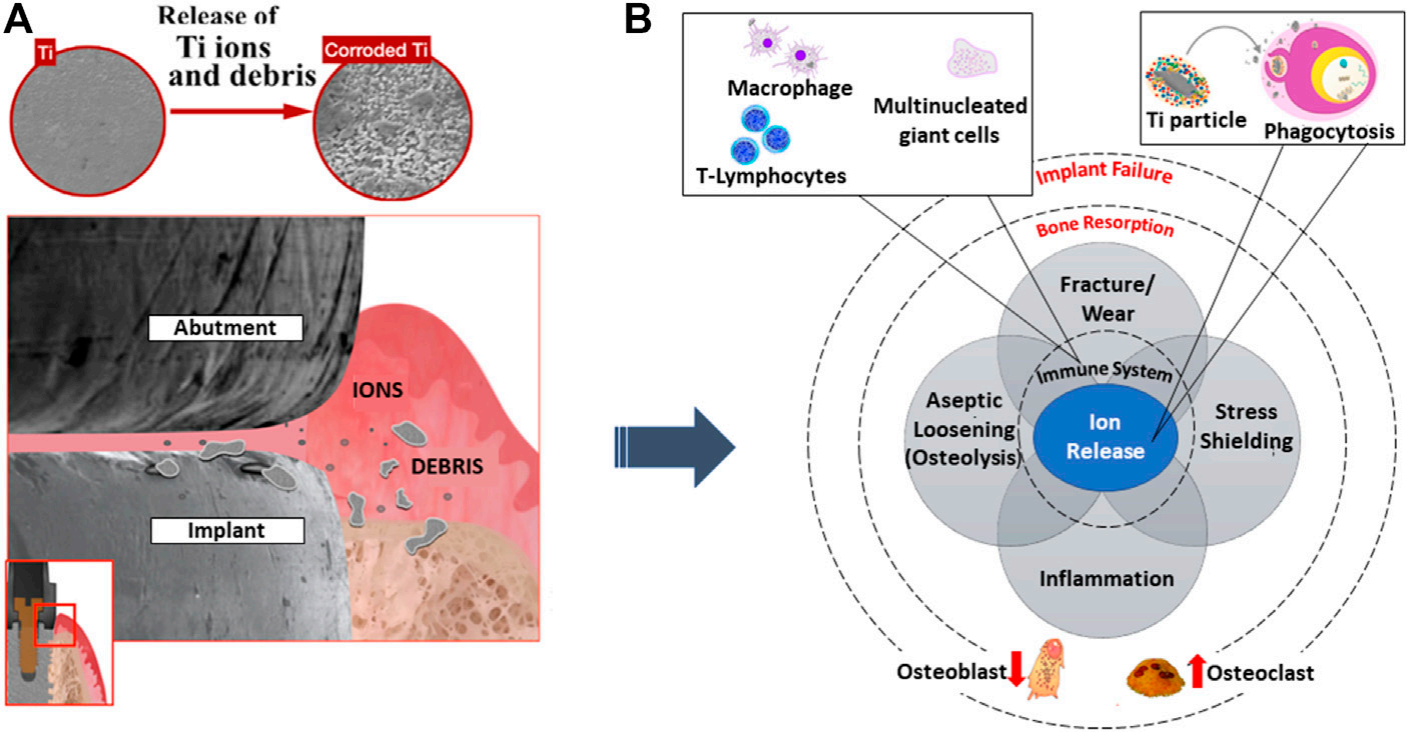
**(A)** The start of foreign ions leaching, adversely affecting **(B)** immune response activities leading to bone resorption and implant failure. Image remodeled from Souza et al. (2020).

Metals are known to corrode easily but some are more resistant. This is due to the formation of a passivation layer that hinders corrosion from taking place. In general, most metals have low corrosion resistance when compared to noble metals such as gold and silver. When in contact with tissue, due to the oxygen diffusion limit contributed by the fast leakage of metal ions in the body, these ions create a high toxicity level, thereby causing adverse effects to the cells (Steinemann, 1998), as shown in Figure 3A. Corrosion is a common phenomenon and can be avoided through surface oxide modification. A study was conducted to identify cell proliferation between bare titanium alloy and cobalt chromium alloy against hydroxyapatite-coated metallic alloys. It was shown that there was a significant increase in cell viability when being coated (Yuan et al., 2018).

**FIGURE 4:**
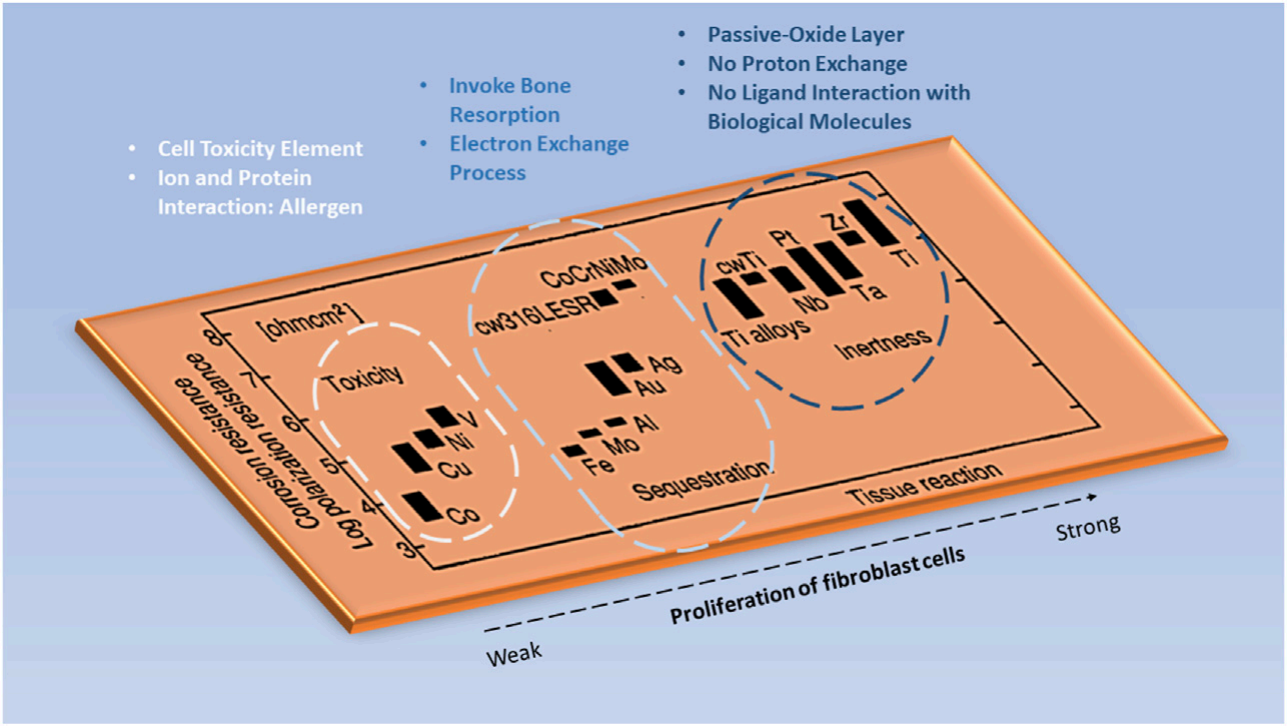
The tissue responsive effect on various metallic elements in the implant. Image remodeled from Steinemann (1998).

When an implant to the body is inevitable to save one's life, one needs to seriously examine the possibility of an adverse reaction of the implant to the body. Figure 4 shows the tissue responsive effect on various common metallic implant materials. It was evident that metallic implants such as cobalt (Co), titanium (Ti) alloys, and iron (Fe) show a relatively similar polarization resistance. Yet, the contrast within tissue destruction is clear for cobalt-based materials. Depending on the implant application, careful consideration must be taken into account for any element that reacts adversely to the human body, since corrosion is also an atomic process and there is a risk of toxicity flowing into the bloodstream (Steinemann, 1998).

Stress shielding is a common phenomenon that takes place when stiffness of a metallic material exceeds the bone mechanical ability. This induces bone resorption which prevents bone growth in accordance with Wolff's law. One way to maneuver away from this issue is by designing a solid implant into the porous architecture (Carpenter et al., 2018). Titanium alloy can be fabricated into porous architecture especially for dental implants, while still maintaining its physiochemical properties thereafter, inducing deep bone integration in the implant (osseointegration) rather than just an interfacial bond. Consequently, avoiding stress shielding as a whole prevents future implant failure (Pałka and Pokrowiecki, 2018).

In addition, titanium is known to be dangerous for casting due to metal fuming and internal oxidation processes. Hence in the traditional way, it is fabricated and machined into the desired shape of an implant (Lütjering and Williams, 2007). Through 3D printing, the mechanical aspect of titanium (low modulus, high specific strength, and low density) can be preserved leading to biocompatibility (Matassi et al., 2013). However, pure titanium is normally avoided in bone implants for load bearing due to its low yield and tensile strength (Pałka and Pokrowiecki, 2018).

Implant failure can be due to several reasons: aseptic loosening, wear, and bacterial infection (Kong et al., 2018), as illustrated in Figure 3B. A study was conducted by Sailer et al. (2009) gathering the percentage of metal components undergoing failure or complication. It was reported that close to 50% was caused by fracture and 24% was due to corrosion. Therefore, emphasis on early surface treatment of implants is ideal to promote successful implantation to the biological system. For example, coating such as a titanium nanotube has an antimicrobial effect which promotes osteoblast formation on the implant surface (Wang and Tang, 2019). Since a titania surface exhibits bioinert behavior when surrounding a biological environment, the physical and chemical characteristics must be restructured. The formation of nanotubular structures promotes strong mechanical interlocking to bone cells as compared to a microstructure surface. As such, the key cellular activities progress while bacterial adherence declines. The inhibition of bacteria is crucial which can result in antibiotic resistance through biofilm formation. In addition, it is reported that through the fabrication process of specific nanotubes with diameters of 30 and 80 nm, a rough surface and low water contact angle are established, eventually aiding cell growth. Likewise, the chemical compositions of oxygen and fluorine are shown to induce both cell adherence and antibacterial ability (Peng et al., 2013).

## Interfacial Bond: From Micro-Cellular to Macro-Metallic Substrates

**FIGURE 5:**
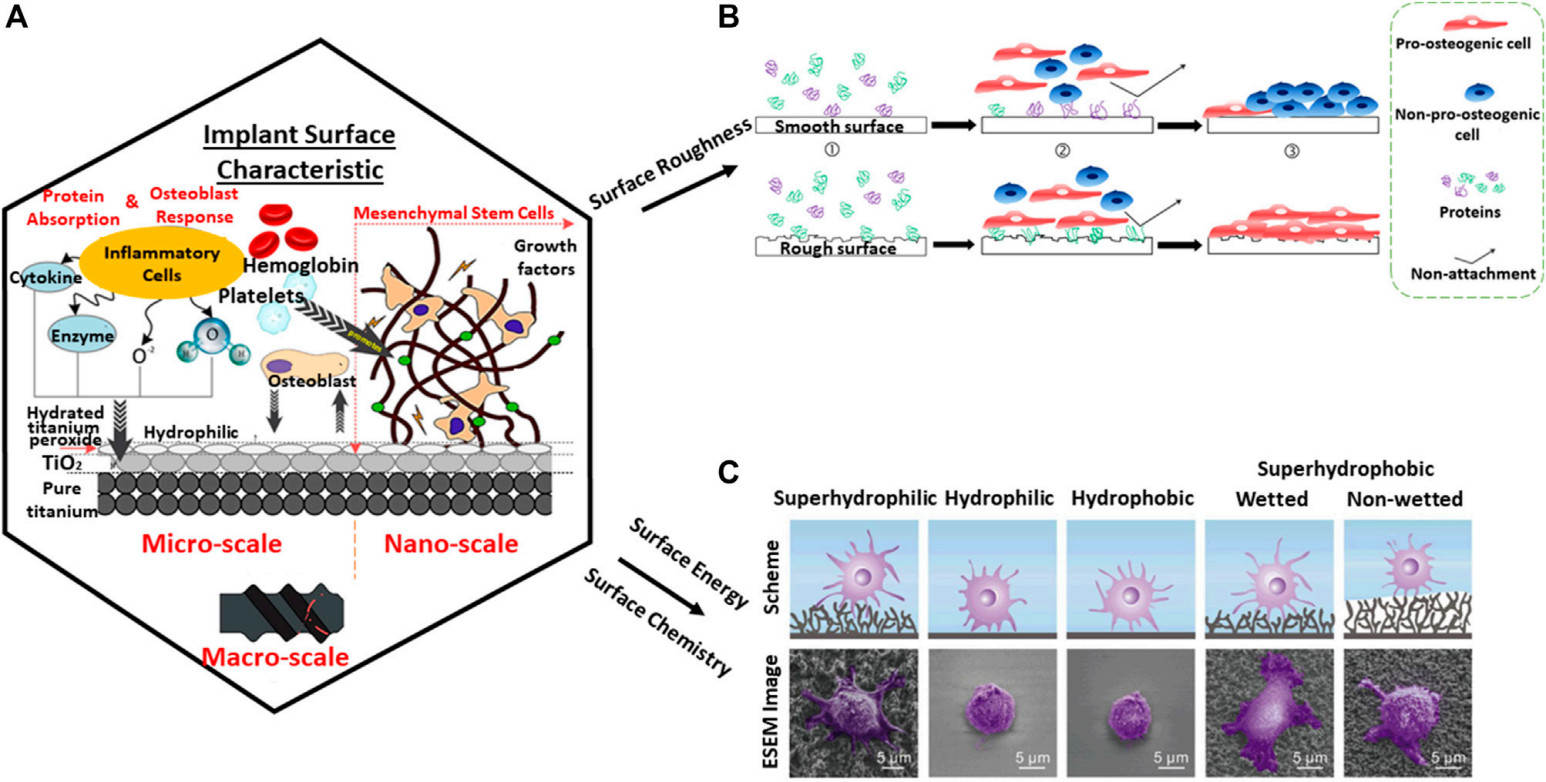
Protein adsorption on the substrate from **(A)** nano-to-macro level which is dependent on several factors. **(B)** Surface roughness (Stich et al., 2021). **(C)** Surface chemistry and surface energy (Meng et al., 2017). Diagram adapted and adjusted from Alipal et al. (2021).

When it comes to medical device design, it is imperative to consider the interaction of the material with the body system at the nano level as shown in Figure 5A. The process of osseointegration involves several complex chains of events. During the initial implantation stage, inflammatory cells such as monocytes, lymphocytes, and granulocytes of the white blood cells (WBCs) first arrive to aid in the healing process around the wound site as observed from a microscale perspective. The major constituents of the blood are plasma, platelets, red blood cells, and WBCs. Many proteins from the blood which are associated with the host inflammatory response, interact with the implant surface when released. However, cells require an intermediate layer to induce successful cell attachment on the implant surface. At the nanoscale, this layer consists of adsorbed water molecules, followed by protein and lipid receptors from the blood, promoting cell attachment on the implant surface. Similarly, the blood platelets release molecules who facilitate formation of fibrin clots which induce migration of mesenchymal stem cells (MSCs). The MSCs have a self-renewal capability which can be differentiated into specialized cells, for example, osteoblasts for bone formation (Gittens et al., 2014). In short, protein adsorption plays a primary role in signaling cell attachment whereby active inflammatory cells such as monocytes, lymphocytes, and granulocytes are actively engaged in the healing process (Onuki et al., 2008), thereafter aiding in the whole bone mechanism of osteoconduction, osteoinduction, and osteogenesis activities (Albrektsson and Johansson, 2001).

The surface of smooth implants has been studied and exhibits an anti-adhesion bond with biological tissue, in contrast to porous implants with better osseointegration capability (Zhang et al., 2014). However, porous implants are complex to fabricate. By conventional methods, fabricating implants of intricate pore structure is challenging due to manufacturing limitations. This can arise from inconsistent dimension tolerance, cost, and time. Likewise, pre-shaped implants may not suit every anatomical model of the patient, hence also causing uneven stress distribution leading to implant failure (Takemoto et al., 2005). Consequently, 3D printing becomes handy in tackling this gap and building a bridge between manufacturing and clinical trials.

### Physiochemical Properties of Implants

Surface roughness, topography, wettability, and chemical composition are crucial to stimulate biocompatibility at the cellular level and interfacial bonding to the metal substrate (Tiainen et al., 2019). Surface roughness is an integral part of a material to allow bone and material interfacial bonding growth by interlocking cells to a material. It is widely known that a smooth surface provides poor stability to an implant as observed in Figure 5B. With surface roughness, osteoblast adsorption is observed which inhibits the osteoclast signal. As a result, full bone deposition is easily observed on the surface (Zhang et al., 2014). However, when it comes to high load-bearing application, surface roughness may pose an issue leading to high cycle fatigue. Osteolysis can occur when bonding strength within implanted bodies loosens thereafter leading to toxic ions leaching, especially when it comes to metallic elements such as cobalt (Wang et al., 2017).

Moreover, due to the lack of natural bone constituents such as bioglass and hydroxyapatite (HA) which form good chemical bonding to the bone, a metallic titanium implant in contrast has poor osseointegration and osteoinductive properties (Steinemann, 1998). It is also worth noting that despite bioceramic materials having similar bone constituents, it does not mean that they will form interfacial bonding with the bone. The key influence is heavily reliant on surface topography—the presence of surface pores hence allowing interlocking of bone cells (Davies, 2007). Similarly, a nonbonding biomaterial such as titania surfaces have the ability for bone bonding (Takatsuka et al., 1995). In hindsight, medical implants must be paired with both microstructure and macrostructure properties to achieve successful tissue integration. The surface chemical-wettability and surface energy of a contact angle below 90^°^ at the microlevel allow for better protein adsorption and subsequently cell attachment (Figure 5C). From the macro-level, the pore-related parameters provide pathways for vascularization and space for bone tissue growth (Song et al., 2019).

Despite 3D printing having multiple prospects, the primary use of 3D printing over traditional manufacturing methods, especially in the medical field, is the ability to print complex lattice structural cells in precise dimensions (Wang et al., 2017). It has been mentioned that a topological feature acts as a stress distributor influencing the mechanical capability by withstanding loads; in particular, the octet truss design has shown superior results compared to other cellular structures (Parthasarathy et al., 2011), whereas triple periodic minimal surfaces promote trabecular bone simulation (Fantini et al., 2017).

### Structural Properties of Implants

Porosity, pore interconnectivity, pore shape, and pore size are essential aspects for the functionality of a built implant, influencing the success rate of 3D printing in a biological system. Porous titanium alloy has a comparable Young's modulus to bone which does not trigger bone resorption as compared to other metallic implants. It is reported that poor bone ingrowth leads to implant loosening, henceforth tackling osseointegration bonding between bone tissue and an implant is vital (Wang et al., 2017; Pałka and Pokrowiecki, 2018). To achieve successful implantation, porous metallic implants should rejuvenate the function of the bone and promote regeneration of the damaged tissues. This is possible by establishing biocompatibility of materials with the living organism, adequate mechanical properties for load-bearing applications, and avoidance of stress shielding, since porosity influences compressive strength and elastic modulus which aid key cellular activities, such as cell adhesion, proliferation, and differentiation (Hrabe et al., 2013).

Although porosity is crucial, it is worth noting that bone ingrowth can be hindered without interconnected pore capability. With porosity, bone cells can penetrate, adhere, and encapsulate the pore structure to provide strong bone mechanisms (Takemoto et al., 2005). Adequate porosity with interconnecting pores allows formation of vascularization as a transport pathway for nutrient diffusion and metabolic waste, which help functional key cellular activities and tissue survival (Karande et al., 2004; Pałka and Pokrowiecki, 2018).

High surface-area-to-volume ratio, pore shape, and pore size are essential for cell attachment and growth. In a study by Mankani et al. (2001), it was observed that HA particles of 100–250 µm showed astounding bone growth, yet those smaller or larger than the critical size tended to show decreased bone formation. Interestingly, it was also noted that flat-sided particles showed no bone formation. It was also concluded that it was unclear how particle size and pore size could be interrelated, but it is hypothesized that the space between the particles will act as a gap indicating that pore size plays a role within bone formation. Subsequently, with the success of controlling printing design for pore sizes and shapes, 3D printing has provided flexibility for designers and engineers to adapt the mechanical behavior of bone tissue accordingly (Hrabe et al., 2013).

**FIGURE 6:**
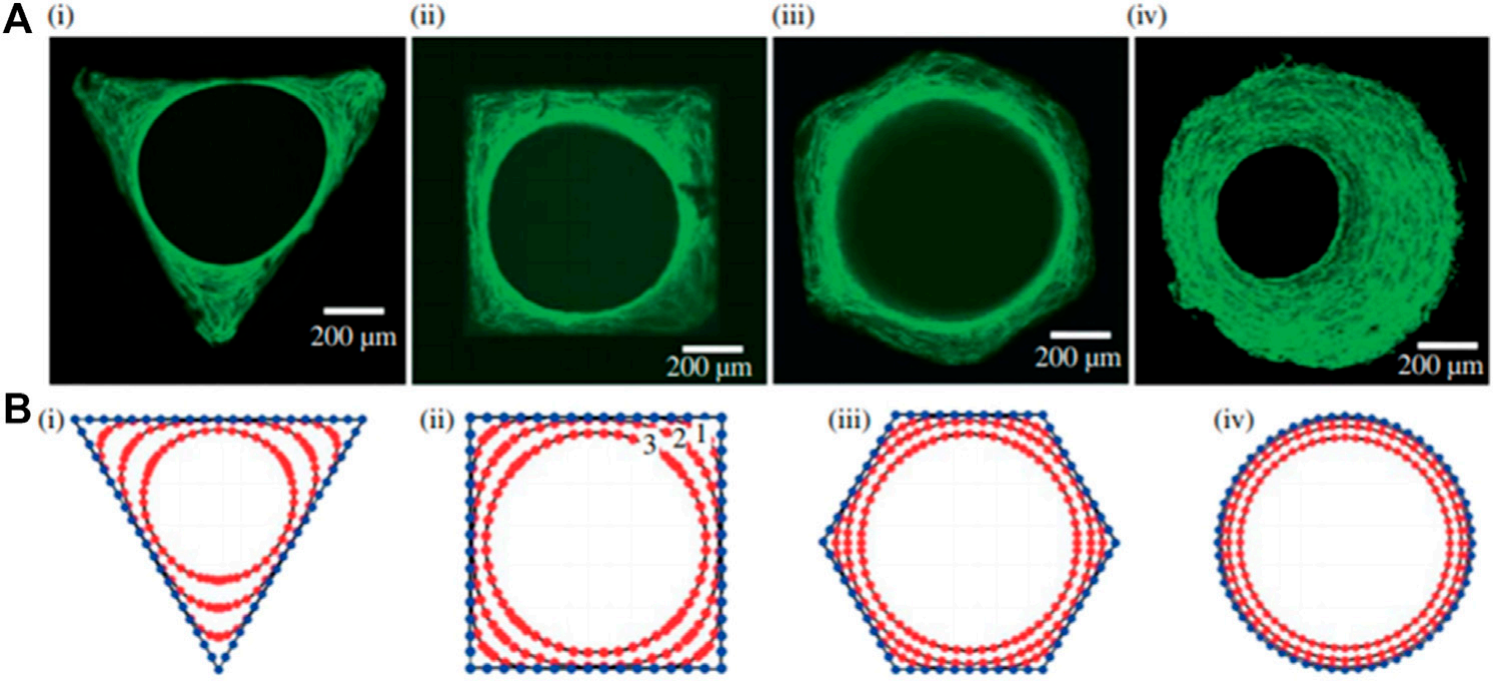
A study on cell interaction and cell-growth concentration as pore shape changes. Reproduced from Rumpler et al. (2008).

In an article by Rumpler et al. (2008), pores of high curvature (rounded corners) displayed more tissue growth as shown in Figure 6. The growth is reasoned by cell–cell neighboring interaction arising from mechanical forces, stimulating physical surface tension. High cell concentration is realized at the surface of high force. Although there is more work to be done to prove the research work, multiple articles have concluded or observed cell proliferation at the curvature as compared to other areas.

## Surface Modification Techniques

Both surface treatment and bioactive coating have been extensively studied for their tribological properties in implants and have been recommended for use in the orthopedic field. While 3D printing allows fabrication of porous architecture, a challenge is faced in tuning surface properties to attain osseointegration with the bone, especially in metallic implants where commonly used materials such as titanium alloy are bioinert (Hwang and Choe, 2018). The release of toxic ions (Al and V) in the titanium alloy poses an actual risk to the biological system, and it is therefore essential to have a dense coating to hinder any ion leakage. Since the benefit of the titanium alloy outweighs the risk, by fine-tuning the material through the formation of a dense oxide film on its exterior or additional surface modification, an unfavorable reaction from the as-printed 3D printing products can be prevented (Song et al., 2019).

### Surface Treatment

The commonly used surface treatment techniques for bio-implants such as micro-arc oxidation (MAO), laser surface texturing (LST), chemical etching, and alkali–heat treatment with HA electrochemical deposition are introduced in the Surface Treatment section, and a summary of their advantages and limitations is shown in Table 1, for each surface treatment method adopted.

**TABLE 1:** Summary of the bioceramic coating method and its limitation.

Method	Material	Process	Advantage	Limitation	Reference
Micro-arc oxidation	Ti6Al4V	Electrodes: Ti/stainless steel	Homogenous oxide film layer, antibacterial and bone-forming cells capability	A few microcracks	Xiu et al. (2016)
		Electrolyte: 0.065 M calcium acetate, 0.03 M monosodium phosphate, 0.065 M EDTA-2Na, 0.5 M sodium hydroxide, bath temperature at 40°C			
		Voltage: 350 V for 5 min			
	Ti-xNb where x = 10, 30, and 50 wt%	Anode: Ti-xNb alloys	Nb content has better biocompatibility, eliminating allergic reactions and bone resorption. Mean porosity increases with increasing Nb content		Kaseem and Choe (2019)
		Electrolyte: 0.15 M calcium acetate and 0.02 M calcium glycerophosphate	Ti-30Nb: Highest corrosion resistance, Ca/P ratio and HA-forming capability		
		Voltage: 280 V for 3 min			
Laser surface texturing	Ti6Al4V	Uniform ridge and groove widths are micropatterned on the sample surface	Swift and efficient process. Reduction in wear/friction and contamination. Superhydrophilicity surface and good mechanical fixation which prevent osteolysis	Tissue response and clinical trial vary on pattern dimension and material selection	Tiainen et al. (2019) Shivakoti et al. (2021)
		Operating machine: SISMA OEM plus 6 W			
		Laser: Q-switched diode-pumped Nd:YAG			
		Laser beam: Circular Gaussian shape profile Spot size: 39 µm			
		Focus beam: 160 mm f-theta objective			
		Input aperture: 10 µm			
Chemical etching	Ti6Al4V	Immersed in 1 ml hydrofluoric acid and 50 ml H_2_O solution, for 2, 5, and 10 min. Posttreatment: immediate cleansing with ethanol and ultrasonic bath	Remove unmelted powder residues in the 3D-printed part	Osseointegration capability only when paired with other methods	Song et al. (2019)
Alkali–heat treatment + HA electrochemical deposition	Ti6Al4V	Performed in 5 mol L^−1^ NaOH solution for 1 h at 60°C	Increase surface area for protein adsorption, induce HA nucleation sites, and good cytocompatibility	Decrease in adhesive strength due to loss of surface structure during alkali treatment	Song et al. (2019)
		Heat-treated for 1 h at 600°C with heating rate 5°C min^−1^ furnace-cooled			
		HA-electrochemical deposited parameters:			
		Electrodes: Pt/Ti/saturated calomel			
		Electrolyte: 2.5 mM calcium chloride hexahydrate, 1.5 mM ammonium dihydrogen phosphate, 0.15 M sodium chloride, bath temperature at 85°C			

#### Micro-arc Oxidation

One method of treating the surface of bio-implants is through MAO, which is also known as plasma electrolytic oxidation. The MAO process relies on an electrochemical method undergoing high temperature and pressure produced by a discharge arc on the metal substrate. Subsequently inducing redistribution of the porous layer and repassivation to a fully formed passive oxide film (Liu et al., 2015). An experimental study indicated that silver-incorporated titanium oxide (TiO_2_) coating through MAO can exhibit antibacterial capability and bone-forming cells (Lv et al., 2019).

**FIGURE 7:**
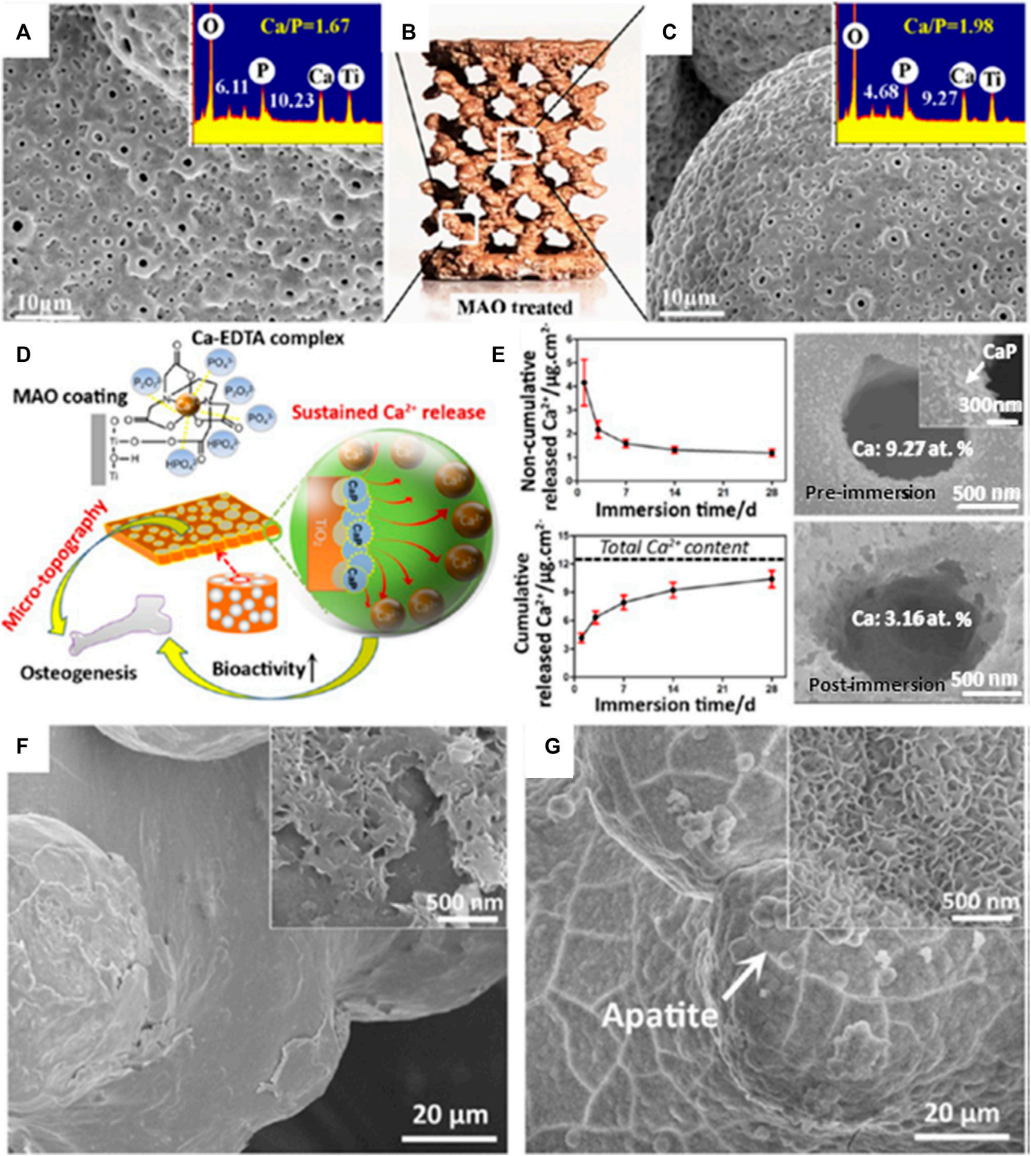
Computer-aided design (CAD) model of multiplanar hexagonal unit cell structures with a macroscopical view of an MAO-treated scaffold **(B)**; the MAO-treated scaffold at the **(A)** outer surface and **(C)** central surfaces. **(D)** The illustrative diagram shows the simultaneous generation of microporous topography and bioactive elements on the macroporous scaffold by MAO treatment in a Ca-Ethylenediaminetetraacetic acid (EDTA)–containing electrolyte and **(E)** the noncumulative and cumulative release curves of Ca^2+^ in phosphate-buffered saline (PBS) for 28 days; scanning electron microscope (SEM) images of the implant surface before and after immersion for 28 days are shown on the right side. **(F)** SEM image of the untreated porous Ti64 scaffold immersed in stimulated body fluid (SBF) for 14 days and **(G)** SEM image of the MAO-treated scaffold immersed in SBF for 3 days. Image reproduced from Xiu et al. (2016).

In a study by Xiu et al. (2016), MAO-treated titanium alloy was adopted for a dual functionality purpose; one where a layer of microporous TiO_2_ is achieved, while another for converting the bioinert titanium alloy into a bioactive surface. A one-step MAO process was applied to a 3D-printed porous Ti6Al4V scaffold to endow the scaffold with a homogeneous layer of microporous TiO_2_ and significant amounts of amorphous calcium phosphate. MAO exhibits a high efficiency in the enhancement of osseointegration of porous Ti64 via optimizing the patterns of bone ingrowth and bone/implant interlocking as seen in Figure 7. Therefore, posttreatment of 3D-printed porous titanium alloy with MAO technology might open up several possibilities for the development of bioactive customized implants in orthopedic applications. It was also noted that the 1 µm micropore between the implant and bone surface acts as an anchorage to bone bonding. Several articles have confirmed the possibility that diameter size plays a critical role in aiding natural bone remodeling (Davies, 2007; Davies et al., 2014) in contrast to a flat surface.

#### Laser Surface Texturing

The use of LST technology has been recommended in recent years, especially within biomedical applications due to its swift speed process, high efficiency and flexibility, its ability to reduce wear and friction, better mechanical fixation, less contamination from unwanted direct contact, and low cost (Earl et al., 2016; Grützmacher et al., 2019; Rosenkranz et al., 2019; Tiainen et al., 2019). In LST, the high-energy beam creates continuous melting and vaporization on the material, aiming at strengthening its tribological behavior (Shivakoti et al., 2021).

For medical implants, crosshatched micro/nano patterns were performed for vascularization and bone adhesion. In a study by Tiainen et al. (2019), a groove-like structure was designed on the surface that showed possibility to optimize the degree of roughness. The process allowed reproducibility, few defects, and good structural integrity which prevents osteolysis. In addition, osteoblast adhesion from superhydrophilic surfaces was observed through a process of laser texturing on the titanium alloy, which indicates surface chemistry and topography integration (Coathup et al., 2017). Overall, these parametric influences are necessary in LST to ensure better compliancy between surface properties and adherence toward biological systems (Shivakoti et al., 2021).

#### Chemical Etching

A previous study has shown that the 3D-printed implant (*via* selective laser melting) has a negative consequence due to unmelted powder residues formed on the printed implant surface. The adverse effect can be detrimental especially for titanium alloy containing toxic ions which may lead to further implant loosening and inflammation through osteolysis. As such, surface treatment by hydrofluoric acid chemical etchant removes toxic unmelted residual powders and even reveals a superior quality surface. The depletion of the oxide film on the titanium surface was also observed when chemically etched but was immediately rebuilt on the substrate, thus providing excellent biocompatibility and corrosion control. Hence, chemical etching is a superior surface treatment method when it comes to removing unmelted powders compared to other common methods such as sandblasting treatment (Song et al., 2019).

#### Alkali–Heat Treatment and HA Electrochemical Deposition

**FIGURE 8:**
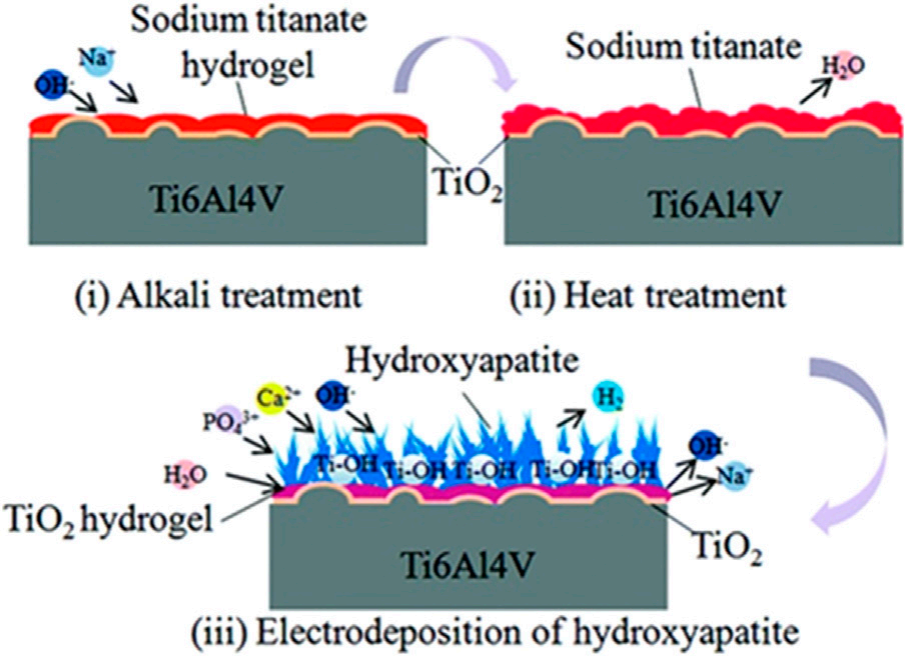
Formation of titanium alloy coating through alkali–heat treatment and HA-electrodeposited surface treatment. Image reproduced from Song et al. (2019).

The bioinert titanium alloy induces fibrous tissues surrounding the implant in contrast to the bioactive implant. The schematic diagram of a multilayer uniform bioactive coating in Figure 8 shows remarkable outcomes in cell cytocompatibility and cell adhesion and proliferation (Song et al., 2019).

Through subjecting implants to alkali–heat treatment, an increase in specific surface area was observed on the surface, which causes accumulation of protein adsorption forming better cell integration. The final process involves electrodeposition of HA acting as nucleation sites on the surface.

### Bioactive Coating

**FIGURE 9:**
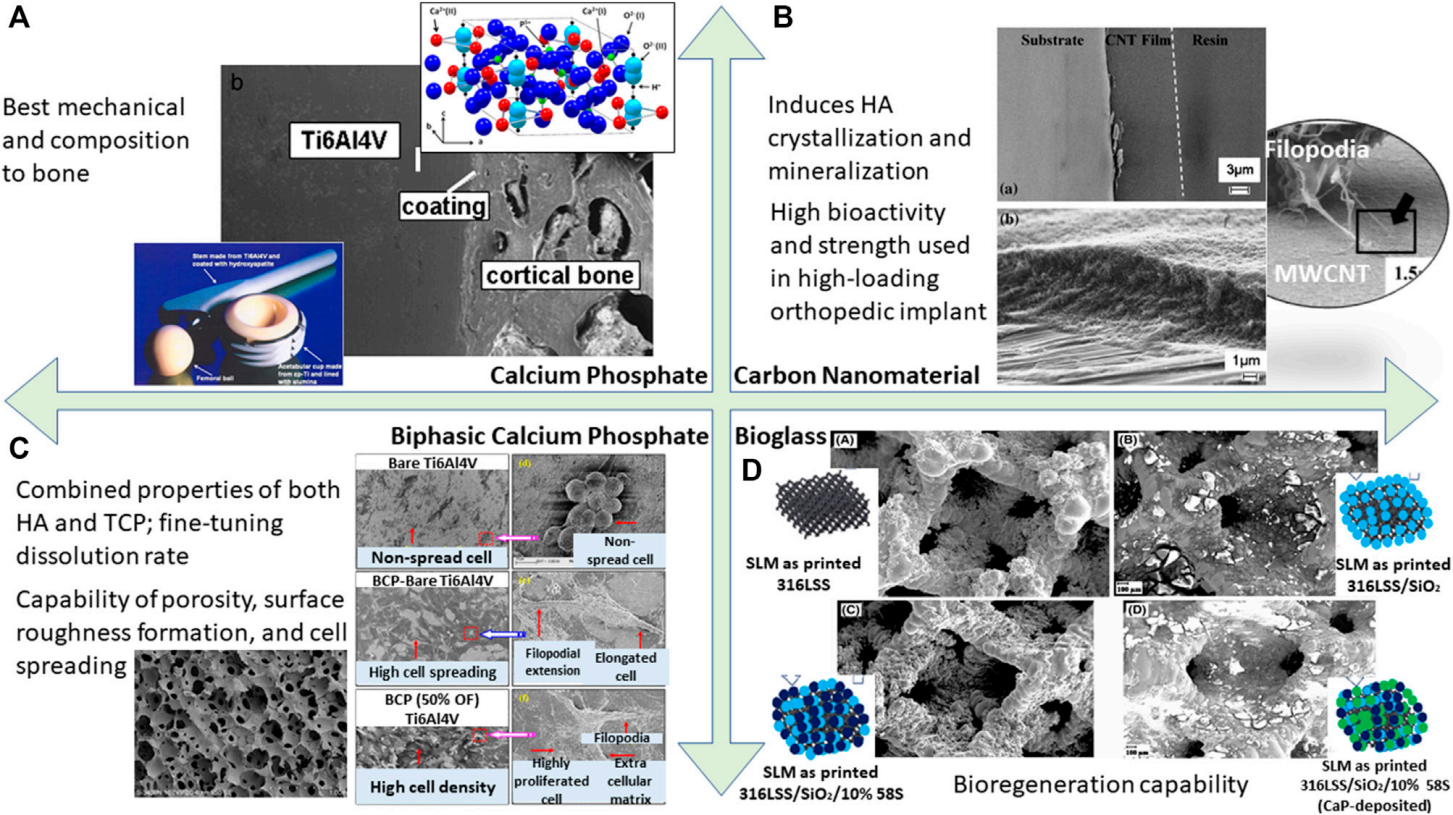
A summary of bioactivity assessment conducted on different types of bioactive coating resulting in cell adhesion capability; **(A)** calcium phosphate (Heimann 2013). **(B)** Carbon nanomaterial (Li et al., 2011). **(C)** Biphasic calcium phosphate (Ebrahimi et al., 2017; Behera et al., 2020). **(D)** Bioglass (Tabia et al., 2021).

Generally, bioceramic is not utilized for load-bearing applications due to its brittle mechanical behavior resulting from its ionic charge counterparts in contrast to metallic material. If implant failure occurs, bioceramic may pose catastrophic effects to the internal body. Despite the fact that the bulk of the implant is made from metallic material, the coating layer exhibits a bioceramic component which forms on the metallic surface. Bioceramic has great bone–tissue interaction which demonstrates biocompatibility in the biological system. Some common bioceramics are calcium phosphate, carbon nanomaterial, biphasic calcium phosphate, and bioglass. There are many advantages surrounding bioceramic coating as classified in Figure 9. Furthermore, bioceramic behaves as an excellent source as compared to other implant materials. It is devoid of both toxic ions such as cobalt or chromium and allergic reactions of nickel ions in stainless steel. It also minimizes disease transmission which is commonly observed in allogenic transplantation. Therefore, the intrinsic behavior of bioceramic has a pivotal role in advancing coating applications within the medical field.

#### Calcium Phosphate

Bioactive ceramic coating on a metal implant has been explored over the last decade (Goodman et al., 2013). For materials such as titanium, readily forming a passive layer alone is not enough for tissue integration. For this reason, HA coating has been extensively researched and is known for its ability to match bone mechanical strength, hence preventing osteoclasts, its chemical bonding to bone (Zhong et al., 2015), and has shown clinical success in inducing bone-growth fixation rather than fibrous connective tissue formation (Kienapfel et al., 1999). The HA coating also acts as a bridge to tackle bone-bonding issues by regulating the biological environment (Goodman et al., 2013). However, whether HA undergoes resorption is still controversial, depending on several factors ranging from physical (anatomical location) (Søballe et al., 1999), to biochemical (increased crystallinity reduces the resorption rate but decreases bone ingrowth) (Overgaard et al., 1999), and mechanical instability (prevent dissolution of HA) (Overgaard et al., 1999).

#### Carbon Nanomaterial

In the past decade, carbon nanotube (CNT) material has emerged as a tool for medical advancement such as in orthopedic coatings. Given its thermal and electrical excellence, this enables the material to act as a pathway for electrical signals from the nerve to a diseased location, hence enabling bone healing and tissue regeneration (Li et al., 2011). For metal implants, it was observed that CNT coating induces HA crystallization and mineralization. This allows the implant as a whole to be biocompatible and form good mechanical bonding to the bone (Akasaka et al., 2006). In particular, CNT has been shown to reinforce composite coating, allowing for high load-bearing application especially in the field of orthopedics. It is paired well with bioglass, polymer, and collagen. On a macroscopic level, it shows minimal porosity, hence its overall attributes can be harnessed as a promising engineering method in the field of biomedical application. There are also several studies mentioning the superior nanoscale surface properties acting for better protein adherence and cell tracking (Li et al., 2011). In addition, the fabrication of CNT was achieved at 10 µm thickness through electrophoretic deposition and even shows a strong adherence between the coating and metallic stainless steel substrate, whereas Ti-C forms a partial reaction in titanium alloy. When reinforced with HA, this coating has been shown to have the superior property of high bioactivity and strength, especially in use for high-loading orthopedic implants (Chen et al., 2005).

#### Biphasic Calcium Phosphate

Though calcium phosphate is evidently known to support the bone growth mechanism between the substrate and bone tissue, the distinct calcium phosphate polymorphs—tricalcium phosphate (TCP), biphasic calcium phosphate (BCP), and HA—pose different osseointegration behaviors. The bioresorption of BCP shows a complimentary functional component due to its combined properties from both HA and TCP. Although HA has better stability, it still has a slow absorption rate when compared to TCP. Likewise, TCP also has higher bioreactivity, but its rapid dissolution may be a cause of concern. Therefore, by fine-tuning these two elemental properties, BCP achieves the desired dissolution rate for specific bio-applications (Ebrahimi et al., 2017). In retrospect, BCP has a superhydrophilic surface which is protein-stimulative adherent compared to the other polymorphs which eventually accelerate the cell proliferation capability (Behera et al., 2020). In an experimental study, it was noted that uncoated titanium alloy shows globular cell morphology, indicating less cell spreading on the surface as compared to BCP-coated titanium alloy. When textured with uniformly distributed grains, cells show even better adherence and growth on the BCP-coated surface (Behera et al., 2020).

#### Bioglass

Bioglass is a synthetic material made of silicon dioxide, phosphates, sodium oxide, and calcium oxide, discovered by Larry Hench (Hench and Jones 2015). Bioglass is a phenomenal material showing promising results with tissue integration. When exposed to body fluids, the material turns into a glass surface where nucleation and growth of HA crystals take place, therefore allowing osteoblast fusion into bone regeneration. Simultaneously, it was observed that because of the dissolution of bioglass, it behaves as a growth factor that sends signals to cells, allowing for better integration that is not seen when it comes to foreign materials due to a fibrous capsule surrounding the implant. In a review by Tabia et al. (2021), a 3D-printed lattice structure of a rhombic dodecahedron was formed. It was stated that this structure promotes reduction in stiffness and weight, and the addition of bioglass coating around this structure's framework promotes better bioactivity yielding to increase bone density and better bone remodeling, hence making it an excellent integration for hip implants. Another study shows that a combination of bioglass and Fe_3_O_4_ nanoparticles further enhanced antimicrobial activity. This nanocomposite coating inhibits infection growth and even shows outstanding corrosion protection as compared to bioglass or Fe_3_O_4_ on its own (Singh et al., 2020).

## Summary and Outlook

The advancement of 3D printing has provided many opportunities for designing intricate and porous metallic biomaterial structures. The 3D printing technique has capability to design precise and controlled topologies while maintaining excellent physical, mechanical, and biological properties. These favorable properties can be enhanced by surface treatment and bioactive coating to enable osseointegration and minimize the risk of implant-associated infections. Surface treatment is used to improve surface roughness in attempts to improve fatigue strength and biological response. With the addition of bioactive coating, significant improvement of bone in-growth capability and bone-implant bonding leads to a more rapid and durable osseointegration. In addition, the release of biofunctional ions in the phase lattice should also be considered in the evaluation of bioactive functions.

Within a biological environment, there are many considerations to be taken into account to improve the service lifetime and function of an implant. An articulate structural design of the implant–tissue interface is critical for the implant to be integrated into the body without complications. Thus, extensive understanding of the protein adsorption and cell adhesion processes at the interface between the tissue and implant surface are particularly meaningful for guiding researchers in choosing the correct surface treatment and bio-functionalization techniques. Moreover, the corrosion property of the bioactive coating also plays a critical role in implant failure. The ions or products released from the corrosion process may generate toxicity or even damage tissues. Therefore, bioactive ceramic coating produced by surface treatment, such as the MAO process, has promising biomedical applications due to its high corrosion resistance. Even better, ceramic coating on implants can be tailored according to the biomedical requirement needed, as they are tunable in their compositions and properties through additives modification, for example, addition of Ca, P, Mg, Si, Sr, etc. Future research efforts should be directed to further improve and control their physical and biological properties for bio-functionalized and long-life coatings.
